# Automatic estimation of brain parenchymal fraction in patients with multple sclerosis: a comparison between synthetic MRI and an established automated brain segmentation software based on FSL

**DOI:** 10.1007/s00234-023-03264-0

**Published:** 2023-12-18

**Authors:** Ilyas Yazici, Britta Krieger, Barbara Bellenberg, Theodoros Ladopoulos, Ralf Gold, Ruth Schneider, Carsten Lukas

**Affiliations:** 1 Institute of Neuroradiology, St. Josef Hospital, Ruhr-University Bochum, Gudrunstrasse 56, 44791 Bochum, Germany; 2 Department of Neurology, St. Josef Hospital, Ruhr-University Bochum, Gudrunstr. 56, 44791, Bochum, Germany

**Keywords:** Multiple sclerosis, SyMRI, SIENAX, Brain parenchymal fraction, MRI

## Abstract

**Purpose:**

We aimed to validate the estimation of the brain parenchymal fraction (BPF) in patients with multiple sclerosis (MS) using synthetic magnetic resonance imaging (SyMRI) by comparison with software tools of the FMRIB Software Library (FSL). In addition to a cross-sectional method comparison, longitudinal volume changes were assessed to further elucidate the suitability of SyMRI for quantification of disease-specific changes.

**Methods:**

MRI data from 216 patients with MS and 28 control participants were included for volume estimation by SyMRI and FSL-SIENAX. Moreover, longitudinal data from 35 patients with MS were used to compare registration-based percentage brain volume changes estimated using FSL-SIENA to difference-based calculations of volume changes using SyMRI.

**Results:**

We observed strong correlations of estimated brain volumes between the two methods. While SyMRI overestimated grey matter and BPF compared to FSL-SIENAX, indicating a systematic bias, there was excellent agreement according to intra-class correlation coefficients for grey matter and good agreement for BPF and white matter. Bland–Altman plots suggested that the inter-method differences in BPF were smaller in patients with brain atrophy compared to those without atrophy. Longitudinal analyses revealed a tendency for higher atrophy rates for SyMRI than for SIENA, but SyMRI had a robust correlation and a good agreement with SIENA.

**Conclusion:**

In summary, BPF based on data from SyMRI and FSL-SIENAX is not directly transferable because an overestimation and higher variability of SyMRI values were observed. However, the consistency and correlations between the two methods were satisfactory, and SyMRI was suitable to quantify disease-specific atrophy in MS.

**Supplementary Information:**

The online version contains supplementary material available at 10.1007/s00234-023-03264-0.

## Introduction

Multiple sclerosis (MS), which is considered the most common chronic inflammatory central nervous system disease in young people [1], leads to demyelination in the white and grey matter, which secondarily induces neurodegeneration and resulting macroscopic atrophy of the brain [2]. Previous research has shown that brain volume loss due to the degenerative component of the disease, evolves in all stages of MS starting at clinically isolated syndromes, and progresses in association with the patients’ disability status [3–5]. The clinical importance of brain atrophy is underlined by the inclusion of whole brain quantification in the current No Evidence of Disease Activity-4 criteria (brain atrophy rates < 0.4% per year, besides changes in lesion load) and that new therapeutic concepts in MS increasingly target neuroprotection [6, 7]. Magnetic resonance imaging (MRI) can be used to quantify brain atrophy [8, 9]. However, manual or semi-automatic volumetric methods are time-consuming and require a great deal of expertise. Automated software applications for brain segmentation such as FSL-SIENAX, CAT12, or FreeSurfer not only help to reduce the time required, but are also more reproducible, objective, and comparable than manual methods [10]. Those methods are mostly based on isotropic 3D T1-weighted (T1w) MRI series and have been used in a broad spectrum of research applications. However, such techniques require advanced post-processing strategies, posing significant hurdles for clinical implementation.

Synthetic-MRI (SyMRI) is another fully automatic quantification tool that is based on a dedicated Multi-Dynamic Multi-Echo (MDME) MRI sequence. This single sequence is typically implemented as a 2D axial acquisition with a 4-mm slice thickness at an in-plane resolution of 1 mm. It is processed by the associated software SyMRI (Synthetic MR, Linköping, Sweden) to instantaneously and fully automatically determine brain volumes and the Brain Parenchymal Fraction (BPF; fraction of total brain volume relative to intra-cranial volume). SyMRI can be integrated and operated within the commonly used radiological picture archiving and communication system (PACS), which allows for use in clinical infrastructures. SyMRI has been applied in different research topics, such as paediatric brain development [11], hippocampal sclerosis [12], aging [13], Alzheimer’s disease [14], and MS [15], to study volume changes in the brain.

In the present study, we aimed to compare the fully automated brain segmentation software applications SyMRI and FSL-SIENAX (SIENAX) in regard to their ability to detect brain atrophy in a large sample of patients with MS. SIENAX was used as the gold standard because it has been used similarly for the establishment of a cut-off value for pathologic brain atrophy [16] and in many other studies on MS [17–20]. We addressed the consistency and differences between BPF estimations with a focus on the methods’ sensitivity to detect disease-related differences in brain volumes between patient groups as well as on their associations with disability levels. Furthermore, we performed a longitudinal analysis of BPF measurements in patients with MS comparing SyMRI and FSL-SIENA (SIENA) for a two-timepoint analysis of percentage brain volume changes (PBVC). We predicted that brain quantification using SyMRI would show good agreement and comparable sensitivity with the established SIENAX method.

## Material and methods

### Study population

Patients with MS were retrospectively collected from the clinic database after routine clinical examinations between August 2018 and July 2021. The included patients with MS fulfilled the diagnostic criteria for MS according to the 2017 McDonald criteria [21]. We further included control subjects (CS) with no neurological deficits who underwent routine MRI scans to rule out intracranial pathologies. Their MRI indications were migraine, headache or vertigo. Exclusion criteria were age > 65 years and other intracranial pathologies (e.g., small vessel disease, ischemic or haemorrhagic stroke, hydrocephalus or tumour). The participants received diagnostic MRI scans using a standardized brain imaging protocol. Associated neurological and demographic information included patient disability status (Expanded Disability Status Scale [EDSS]) [22], age, sex, and disease duration; data were extracted from patient files. The study protocol was approved by the ethics committee of the Medical Faculty. Guidelines from the Strengthening the Reporting of Observational Studies in Epidemiology were carefully followed [23].

### MRI acquisition

MRI was performed using a 1.5-T scanner (Aera; Siemens Healthineers, Erlangen, Germany) using a 16-channel head/neck matrix coil. The standardized imaging protocol included conventional contrast-weighted imaging of the brain: sagittal 3D T1w Magnetization Prepared Rapid Acquisition with Gradient Echoes sequence (repetition time, 10 ms; echo time, 4.6 ms; acquisition matrix, 240 × 240; voxel size, 1 × 1 × 1 mm^3^; 180 axial slices) and sagittal 3D Fluid-attenuated Inversion Recovery sequence (repetition time/echo time/inversion time: 5000 ms/332 ms/1800 ms; flip angle, 120°; number of excitations, 1; resolution, 1 × 1 × 1 mm^3^; matrix, 256 × 230; 160 slices). In addition, the axial MDME sequence for SyMRI was used for all participants (Quantification of Relaxation Times and Proton Density by Multi-echo Acquisition of a Saturation Recovery using Turbo Spin-echo Readout with repetition time, 6930 ms; echo time 1, 23 ms; echo time 2, 102 ms; inversion time, 29 ms; acquisition matrix, 256 × 146; voxel size, 1 × 1 × 4 mm^3^).

### Quantitative analysis of MDME sequence with SyMRI

The automatic post-processing software used for SyMRI allows for simultaneous voxel-wise quantification of relaxation rates R1 and R2 and proton density (PD) based on the MDME sequence [24, 25]. Segmentation of the brain into white matter (WM), grey matter (GM), and cerebrospinal fluid (CSF) is based on a tissue look-up grid in a three-dimensional R1–R2–PD space that has been created based on different brain regions in healthy volunteers [26]. SyMRI thus provides fractions of these tissue classes that are in each voxel: GM, WM and CSF. Those voxels that are not categorized as WM, GM, or CSF are summarized as the non-WM/GM/CSF (NON) class. Tissue volumes are calculated via multiplication of the tissue fractions per voxel by the voxel size and subsequent summation across all brain voxels. In addition to maps for each tissue class, SyMRI calculates volumes (in ml) of GM, WM, CSF, NON, and the total brain tissue and volume fractions relative to the total intracranial volume (ICV = GM + WM + CSF + NON). BPF is determined by dividing the sum of GM, WM, and NON by total intracranial volume. In previous publications, excellent scan–rescan reliability of BPF, GM, and WM has been shown in healthy controls and patients with MS [16].

### Post-processing of the 3D T1w datasets

**Fig. 1:**
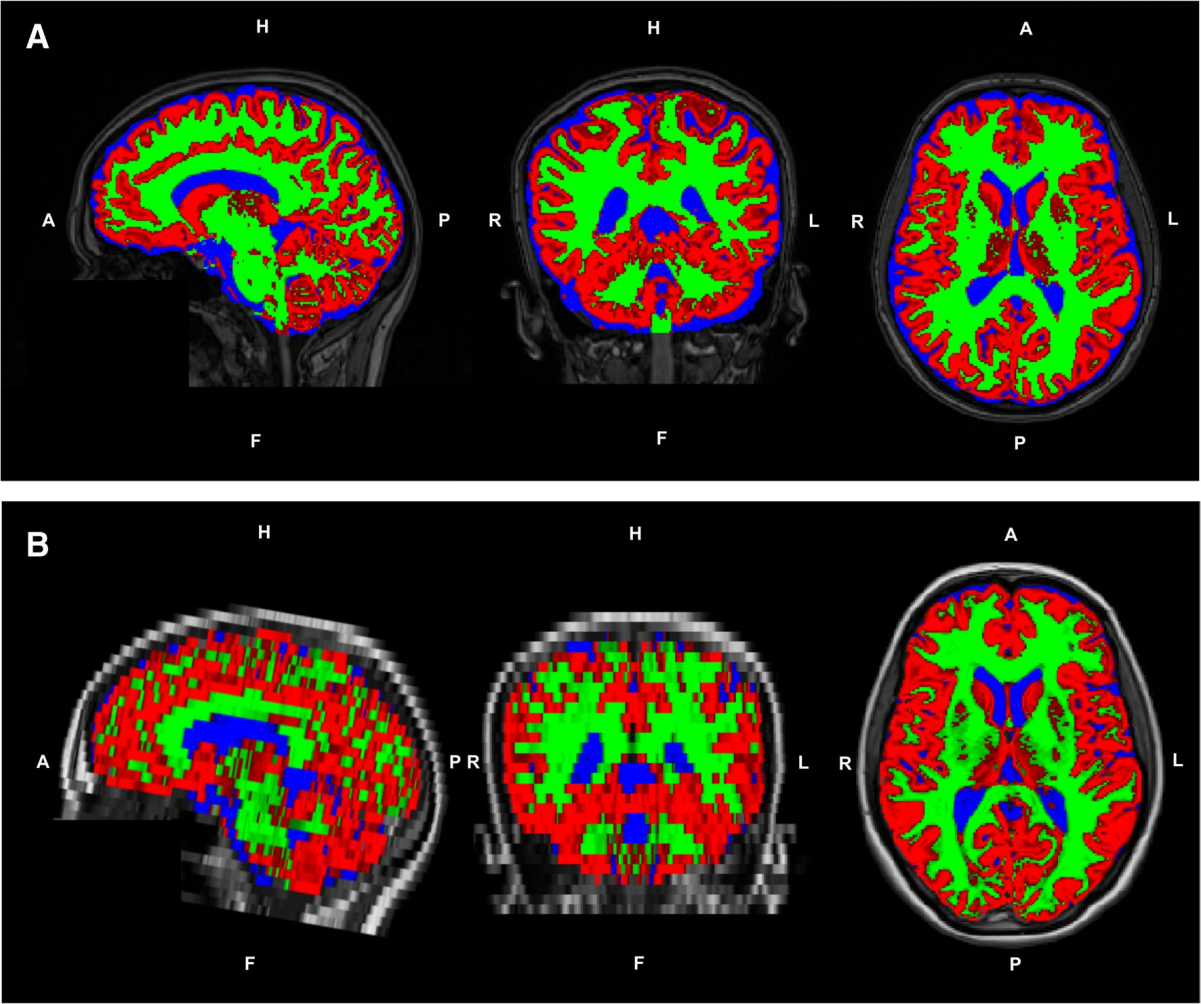
Comparison of tissue segmentation maps of a a healthy participant (female, 36 years old)﻿ generated by SIENAX (A) and SyMRI (B) shown on corresponding T1-weighted images; green overlay: white matter; red overlay: grey matter; blue overlay: cerebrospinal fluid

Prior to brain volumetry, the 3D T1w datasets were first corrected for hypointense lesions using lesion filling based on corresponding FLAIR lesion maps using the lesion prediction algorithm included in the lesion segmentation toolbox (LST toolbox; v2.0.15; http://www.statistical-modelling.de/lst.html.27). To exclude the areas containing the cervical spinal cord, the filled images were cropped using FSL’s robustfov function. Brain extraction was performed using FSL. Instead of the default BET option, a fractional intensity threshold of 0.1 [27] and the option for bias field correction and neck clean-up was used. The images were then processed by SIENAX [28, 29], part of FSL [30], which estimates brain tissue, GM, and WM volumes normalised to subject head size. SIENAX begins by extracting brain and skull images from the single whole-head data [31]. Brain images are then affine-registered to the MNI152 space [32, 33] (using skull image to determine registration scaling); this is primarily done in order to obtain the volumetric scaling factor, which is used in the normalisation based on head size. Next, tissue-type segmentation with partial volume estimation is carried out [34] to calculate the total brain tissue volume (including separate estimates of GM, WM, cortical GM, and ventricular CSF). In order to guarantee comparable methods, only non-normalized volumes were used, and BPF was calculated using the results from FSL. For this, CSF masks were additionally saved using the debugging option in SIENAX, and these were used to estimate CSF volumes using the fslmaths function. BPF was then calculated: (GM + WM) / (GM + WM + CSF + NON). Figure 1 shows an example of the tissue segmentation maps from SyMRI (axial 4-mm slices, MDME) compared to those from SIENAX (isotropic lesion-filled 3D-T1w MRI) in a healthy participant (female, 36 years old).

### Longitudinal analysis

To analyse longitudinal data, the lesion-filled 3D T1w data were processed with FSL-SIENA (SIENA) to obtain the two-timepoint percentage brain volume change (PBVC). SIENA is a registration-based software that uses the apparent displacement of the brain surface edge points in the images at both timepoints to estimate volume changes [31] The PBVC from SyMRI data, and exploratorily from SIENAX results, between baseline (BL) and follow-up (FU) was calculated using a two-timepoint substarction method with the following equation.

$$\frac{BPF(FU)-BPF(BL)}{BPF(BL)}\times 100$$

### Statistical analysis

To examine the agreement, consistency, and differences between SyMRI and FSL, the volume estimates were compared using different statistical analyses. First, differences in BPF and volume estimates of WM (WMV) and GM (GMV) were calculated based on relative and absolute differences, expressed as percentages [35]. Positive values for relative differences reflected higher volumes derived by SyMRI compared to FSL. Relative (rDiff) and absolute (aDiff) volume differences were calculated by

$$rDiff\left(\%\right)=\left(\frac{SyMRI-FSL}{\frac{SyMRI+FSL}{2}}\right)\times 100$$

$$aDiff\left(\%\right)= \left(\frac{|SyMRI-FSL|}{\frac{SyMRI+FSL}{2}}\right)\times 100$$

Second, the consistency of the two methods was assessed using intraclass correlation coefficients (ICCs, Model: 2-way mixed, Typ: absolute agreement), which were interpreted according to the classification by Chicchetti et al. [36]as follows: < 0.4, poor; 0.4–0.59, average; 0.6–0.74, good; > 0.75, excellent agreement.

Third, Bland–Altman plots were created, which showed the differences between volumes measured by the two methods plotted against their mean. This allowed for the identification of systematic biases as well as the amount of variation between the two methods. Fourth, Pearson correlation analysis was performed to assess the correlations between volume estimates. Furthermore, volume differences were examined between the different disease types and control participants included in this study. For this, one-way analyses of variance with disease duration, age, and sex as covariates was performed followed by pairwise Tukey post hoc tests.

To check the effect of lesion filling on GM and WM segmentation results we compared SIENAX derived GM and WM volumes with and without prior lesion filling of the 3D-T1w datasets. For analyses of group mean differences we used paired *t*-tests.

All analyses were intended to compare both methods in a cross-sectional manner. In addition, the agreement between SyMRI and FSL was investigated longitudinally by comparing the PBVCs. Again, Pearson correlation, Bland–Altman plots, and ICC analyses were conducted.

## Results

Images and segmentation maps were visually inspected for image quality after analysis with SyMRI and SIENAX. We excluded 14 patients due to artefacts or incorrect tissue segmentation, which resulted in a total of 244 patients and control participants. In the patients, both MS subtypes were represented: 110 cases of relapsing–remitting MS (RRMS) and 106 of primary or secondary progressive MS (PMS, including *N* = 83 SPMS and *N* = 23 PPMS). The demographic data of the final cohort are summarised in Table 1. Details of the SPMS and PPMS subgroups are shown in the electronic supplement (Table S1). Patients in the PMS group were older and had significantly higher EDSS scores and longer disease duration than patients with RRMS.

**Table 1:** Demographic data of patients and control participants used for cross-sectional analysis

Characteristic	Overall *N* = 244^1^	CS *N* = 28^1^	RRMS *N* = 110^1^	PMS *N* = 106^1^	*p*-value^2^
Age [years]	50 (38, 58)	35 (27, 41)	42 (35, 53)	58 (50, 65)	< 0.001 ^a^
Sex					
Female	158 (65%)	23 (82%)	75 (68%)	60 (57%)	
Male	86 (35%)	5 (18%)	35 (32%)	46 (43%)	
EDSS	5.0 (2.5, 6.5)	NA	2.5 (1.5, 4.0)	6.5 (5.5, 7.0)	< 0.001
Unknown	49 (20%)	28 (100%)	17 (15%)	4 (3.8%)	
Disease duration [years]	11 (4, 22)	–	8 (4, 15)	21 (12, 28)	< 0.001 ^a^
Unknown	3 (1.2%)	–	2 (1.8%)	1 (0.9%)	

^1^Median (IQR); n (%)

^2^Kruskal–Wallis rank sum test; Pearson's Chi-squared test

^a^mean significantly different between CS, PMS, and RRMS (*p* < 0.01, pairwise *t*-test)

*CS,* control subjects; *PMS,* progressive MS; *RRMS,* relapsing–remitting MS

### Comparison of volume measurements

Brain volumes averaged across the whole study sample for SyMRI and SIENAX and parameters for method comparisons are summarised in Table 2. Overall, higher BPF and GMV values were derived from SyMRI than from SIENAX. In contrast, SyMRI estimated lower WMV and CSF volumes (CSFVs) compared with SIENAX. The percentage differences between the two methods were smallest for BPF and higher, at a similar level, for GMV and WMV. The CSFV estimates showed the largest differences > 30%, wherein the between-subject variability of CSFV based on SyMRI was considerably larger than that based on SIENAX.

**Table 2:** Comparison of estimated brain parenchymal fraction (BPF), grey matter volume (GMV), and white matter volume (WMV) and inter-method differences between SyMRI and SIENAX

	BPF	GMV [ml]	WMV [ml]	CSFV [ml]
SIENAX	0.74 (0.04)	576 (71)	530 (58)	395 (64)
SyMRI	0.80 (0.07)	657 (84)	451 (67)	291 (104)
Percentage difference [%]				
Relative	7.6 (4.2)	13 (5.5)	-16 (10)	-35.6 (22.6)
Absolute	7.8 (3.8)	13.2 (5)	17 (10)	36 (22)
ICC	0.652	0.734	0.59	0.636
Correlation coefficient	0.94	0.90	0.75	0.90

Mean (SD); *ICC*, intra-class correlation coefficient

The consistency between the two methods quantified using ICCs was good for BPF and WMV and excellent for GMV. In order to quantify the dependence of measurement accuracy on brain atrophy, we determined the ICCs of patients with low and high atrophy (*N* = 214, high atrophy: BPF < 0.65, low atrophy: BPF > 0.73). We assume that low atrophy refers to high brain volume and therefore high BPF and vice versa. The agreement between SIENAX and SyMRI was better for patients with low BPF (ICC = 0.723, *N* = 111, 95% confidence intervall [-0.11; 0.90]) compared to high BPF (ICC = 0.198, *N* = 103 95% confidence intervall [-0.05; 0.55]).

**Fig. 2:**
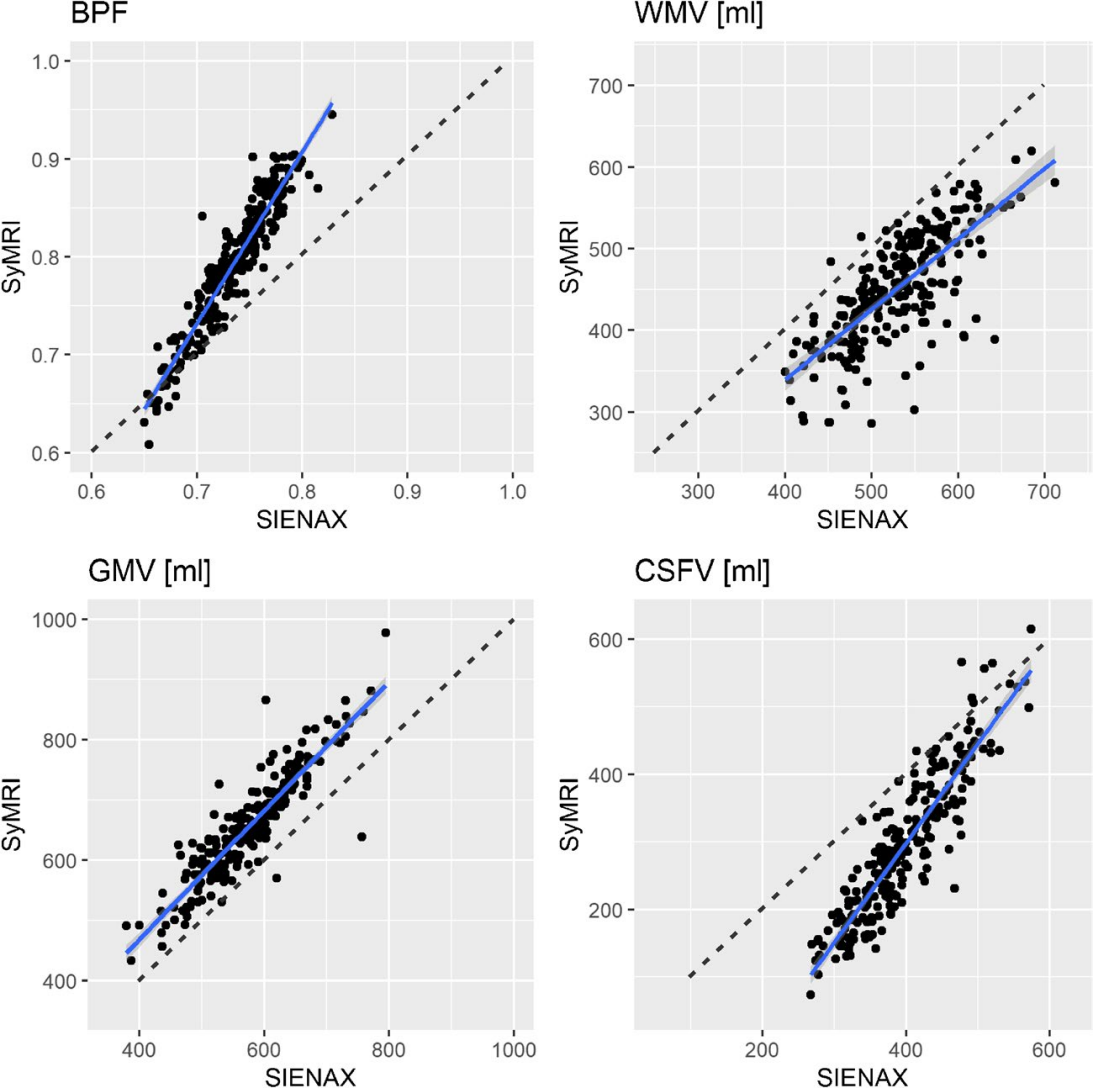
Scatterplots of SyMRI brain volume estimates of all participants plotted against corresponding SIENAX estimates; blue lines: linear regression; dashed line: identity line representing perfect agreement between the two methods. Upper row, left: brain parenchymal fraction BPF, upper row right: white matter volume WMV, lower row left: grey matter volume GMV, lower row right: cerebrospinal fluid volume CSFV

**Fig. 3:**
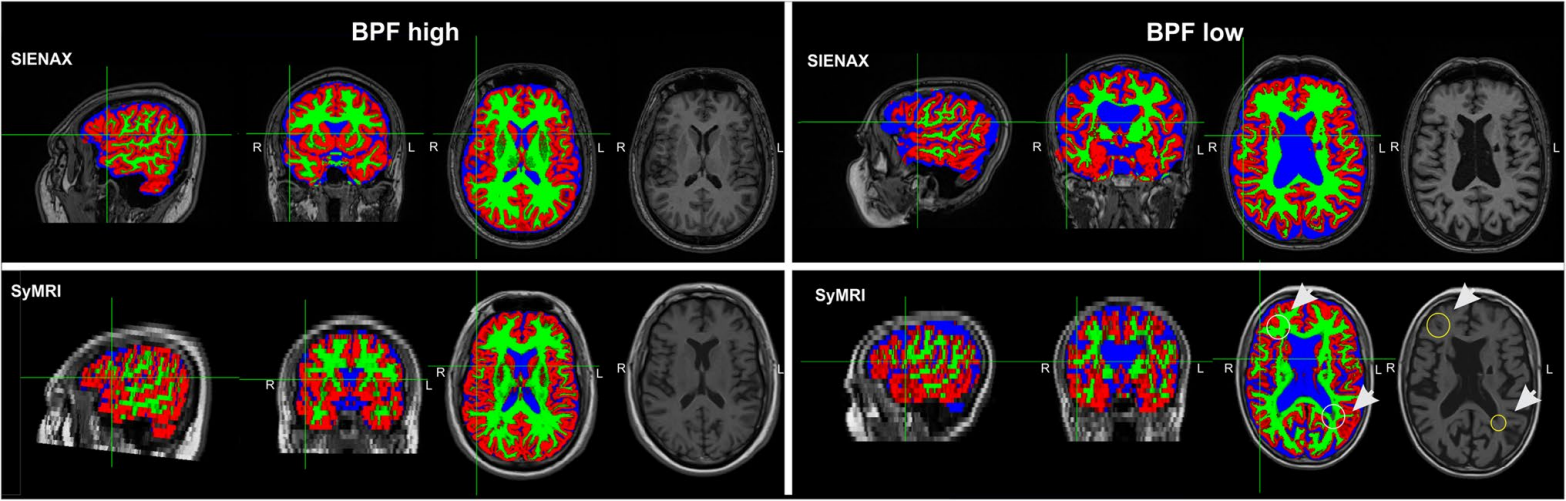
Comparison between brain segmentations with SIENAX and SyMRI in patients with low-grade brain atrophy (left column, termed “BPF high”) contrasted with marked brain atrophy (right column, termed “BPF low”); overlays shown on T1-weighted images represent the segmentation maps of white matter (green), grey matter (red), cerebrospinal fluid (blue); the right most columns show the corresponding T1-weighted images. Circles and white arrows in the right column point at examples of WM lesions in the synthetic T1-weighted images (without lesion filling) which are classified as GM in the SyMRI segmentation maps

Figure 2 depicts the correlations between results derived from SyMRI and SIENAX. For all measurements (BPF, GMV, WMV, CSFV), strong correlations between both techniques were obtained (Table 2; all *p* < 0.001). WMV analyses performed by SyMRI showed higher numbers of outliers with noticeably lower volumes compared to SIENAX. This probably reflects the differences between the methods in how they handle WM lesions, where lesion filling is included in the SIENAX post-processing pipeline, but lesions are partially classified as other tissue classes or NON in SyMRI, leading to a reduction of the WMV. This effect is also visible in Fig. 3 that shows examples of tissue segmentation with SIENAX and SyMRI in a patient with high BPF (low grade brain atrophy) and a patient with low BPF (marked brain atrophy). Here, the deviating use of lesion filling led to differences in WM and GM segmentation, wherein WM lesions were partly classified as GM (marked with arrows in the right colums of Fig. 3. However, the systematic bias between GM and WM volume estimation between the two methods was not mainly induced by the effects of lesion filling. In an additional analysis we compared brain segmentation using SIENAX on the non-lesion filled original 3D-T1w datasets with the default segmentations of GM and WM on lesion filled data. The results, which are presented in the electronic supplement (Table S2 and Fig. S1) showed that the effect of lesion filling on GM and WM volumetry was small with no significant differences of the group mean GM and WM between lesion-filled and and non-filled segmentations. Accordingly, the comparison of GM and WM volume estimation using SIENAX and SyMRI only in the group of control subjects were lesion filling was not necessary, confirmed the systematic bias between the two methods (supplement, Fig. S2). Thus, other effects must contribute to these segmentation differences with larger GM and smaller WM volume in SyMRI than in SIENAX. Potential sources might be stronger partial volume effects in SyMRI due to the anisotropic resolution, misclassification of WM as GM at the periventricular boundary CSF and WM due to partial volume effects, or differences in the contrast between GM and WM between the underlying MRI sequence types shifting the classification boundary between WM and GM.

**Fig. 4:**
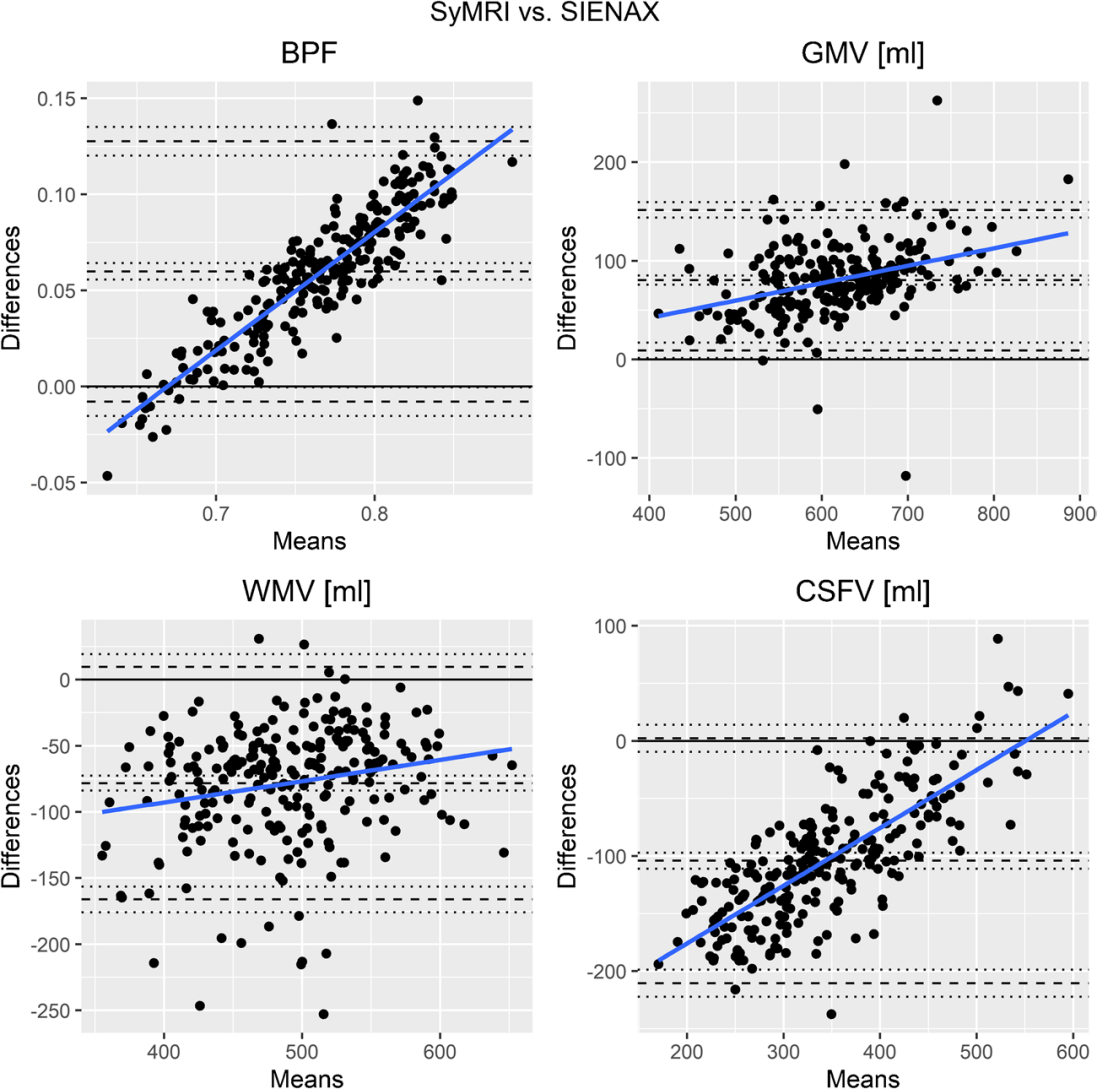
Bland–Altman plots for comparison of brain parenchymal fraction (BPF), grey matter volume (GMV), white matter volume (WMV), and cerebrospinal fluid volume (CSFV) values between SyMRI and SIENAX (differences are SyMRI – SIENAX). Dashed lines: bias between the methods (represented by the mean of the differences between the methods; middle) and upper and lower limit of agreement (+﻿-1.96 standard deviation); dotted lines: 95% prediction ranges of the bias, upper- and lower limit of agreement; bold black line: zero level; bold blue line: linear regression line

In the Bland–Altman plots (Fig. 4), most of the values of BPF, GMV, and CSFV were within the limits of agreement, while WMV showed a small number of outliers with considerably larger differences between the two methods. Overall, the average bias between the methods was about 10% for BPF and GM, higher for WM and considerably larger for the CSFV (about 25%, Fig. 4). Within the limits of agreement, the Bland–Altman plots indicated proportional differences in BPF as the differences increased with larger means. Thus, BPF estimations by the two methods showed better agreement when there was marked brain atrophy than in participants with high brain volumes. Corresponding but less prominent associations were found for GMV. For CSFV, SyMRI measured smaller volumes SyMRI than SIENAX in participants with small CSF volumes (low brain atrophy), while CSFV differences were small in participants with large mean CSFVs (strong brain atrophy). This effect of differences in CSF segmentation is also visible in Fig. 3 where CSF segmentations are similar between SIENAX and SyMRI in a patient with marked brain atreophy (BPF low), while SIENAX showed a considerably smaller CSF volume than SyMRI in a patient without brain atrophy (BPF high).

### Group differences

**Fig. 5:**
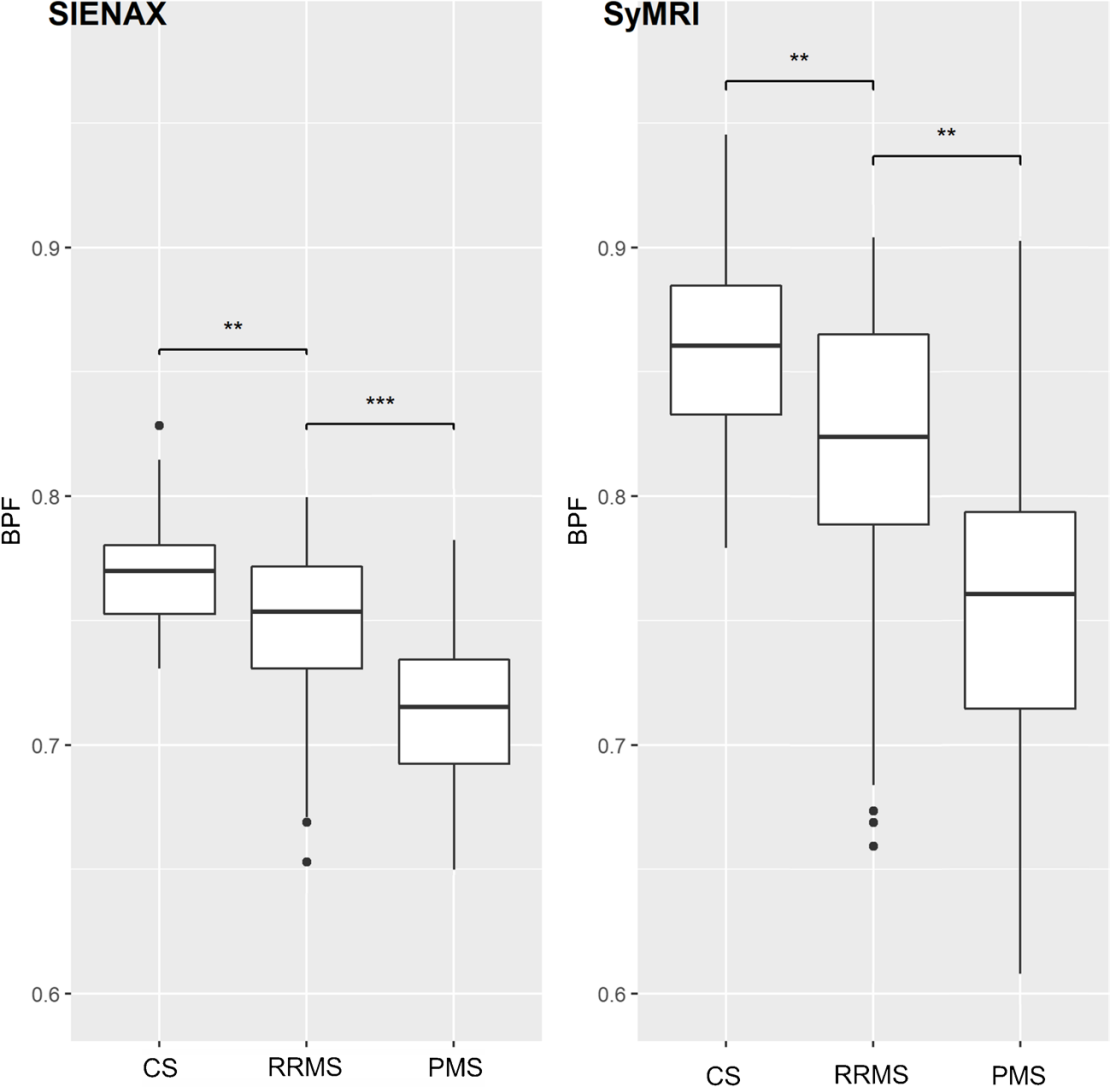
Brain parenchymal fraction (BPF) estimates for control subjects (CS) and patient groups (relapsing–remitting MS [RRMS], progressive multiple sclerosis [PMS]) derived from SIENAX and SyMRI; ** p < 0.05, *** *p* < 0.001, pairwise post hoc tests

To evaluate the suitability of SyMRI for detecting disease-specific brain changes, we conducted comparisons between the RRMS, PMS, and CS groups for BPF (Fig. 5). Analysis of Variance controlling for disease duration, age, and sex as covariates and subsequent post hoc tests yielded similar significant group differences for both SyMRI and SIENAX, namely significantly lower BPF for PMS compared with CS and RRMS (Table 3).

**Table 3:** Post hoc tests for group comparisons of BPF

	FSL				SyMRI			
Comparison	Estimate	CI lower	CI upper	*p*	Estimate	CI lower	CI upper	*p*
RRMS — CS	-0.007232	-0.021383	0.006919	0.445206	-0.012655	-0.039025	0.013715	0.48929
PMS — CS	-0.0243	-0.041614	-0.006985	0.003286	-0.04105	-0.073315	-0.008786	0.00861
RRMS — PMS	0.017068	0.006444	0.027691	0.000602	0.028395	0.008599	0.048191	0.00259

p-values determined using post hoc Tukey tests*;* *MS*, multiple sclerosis; *RRMS*, relapsing–remitting MS; *PMS*, progressive MS; *CS*, control subjects; *CI*, confidence interval

### Correlation with clinical data

Correlations between BPF and clinical data were significant for both SyMRI- and FSL-derived values across all patients. Thus, Pearson correlation between EDSS and BPF revealed negative relationships with coefficients of -0.54 (*p* < 0.001) for both methods. Similarly, disease duration was negatively associated with BPF derived by both methods with a coefficient of -0.56 for SyMRI and -0.55 for FSL (*p* < 0.001).

### Longitudinal analysis

In total, 35 patients (*N* = 12 RRMS, *N* = 23 PMS consisting of *N* = 19 SPMS and *N* = 4 PPMS) received follow-up MRI and were included for a longitudinal analysis with a follow-up interval of 1–2 years. Demographic data of this subgroup are summarised in Table 4.

**Table 4:** Demographic data of patients used for longitudinal analysis

Characteristic	BL, *N* = 35	FU, *N* = 35
MS subtype		
RRMS	12 (34%)	12 (34%)
PMS	23 (66%)	23 (66%)
Age	53 (44, 58)	54 (44, 59)
Sex		
Female	18 (51%)	18 (51%)
Male	17 (49%)	17 (49%)
EDSS	4.50 (3.50, 6.38)	4.50 (2.75, 6.00)
Unknown	1	1
Disease duration [years]	11 (7, 23)	11 (7, 24)
Time between MRI examinations [months]		15 (11, 20)

n (%); Median (IQR)

**Fig. 6:**
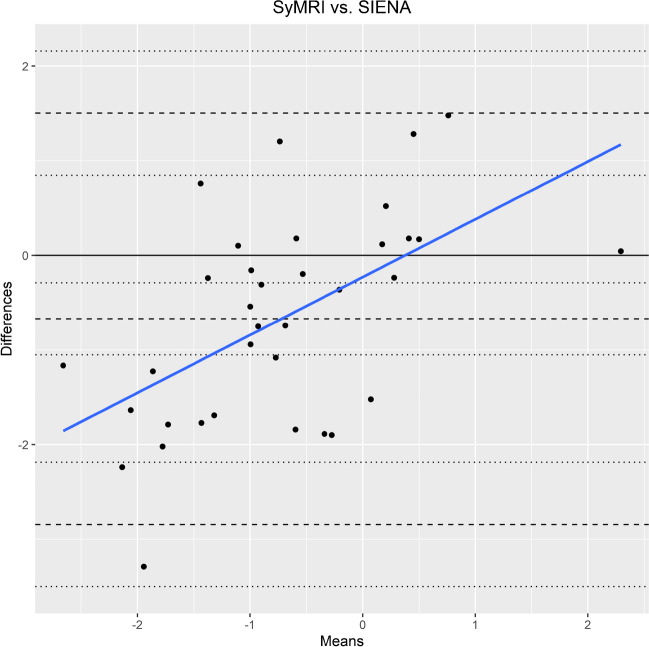
Bland–Altman plot for comparison between longitudinal brain volume changes derived from SyMRI and SIENAX; Differences [%]: SIENA PBVC – SyMRI PBVC; Means [%]: means of SIENA PBVC and SyMRI PBVC; Dashed lines: bias between the methods (represented by the mean of the differences between the methods; middle) and upper and lower limit of agreement (+—1.96 standard deviation); dotted lines: 95% prediction ranges of the bias, upper- and lower limit of agreement; bold black line: zero level; bold blue line: linear regression line

Overall, higher rates of brain volume changes (both absolute and annualized) were obtained by SyMRI than by SIENA (Table 5). Pearson correlation revealed a significant positive correlation between PBVC obtained by SyMRI and SIENA (coefficient: 0.6; *p* < 0.001). The agreement between the two methods was good (ICC = 0.628, confidence interval = [0.149, 0.826]; *p* = 0.009). On the group level, the differences were larger in the PMS groups compared with the RRMS group (Table 5). Although the Bland–Altman Plot (Fig. 6) also showed overall good agreement between the two methods, there was a tendency for increased discrepancy in patients with greater atrophy rates, with larger PBVC values from SyMRI.

**Table 5:** Average percentage brain volume changes (PBVC) obtained by SIENA and SyMRI. Annual atrophy rates were calculated using the time between two MRI examinations

Change rates [%]	Overall *N* = 35	PMS *N* = 23	RRMS N = 12
SIENA PBVC	-0.39 (0.84)	-0.65 (0.68)	0.12 (0.89)
SyMRI PBVC	-1.06 (1.39)	-1.44 (1.16)	-0.32 (1.55)
Annual			
SIENA PBVC	-0.10 (1.09)	-0.47 (0.54)	0.61 (1.50)
SyMRI PBVC	-0.50 (1.52)	-0.85 (0.86)	0.17 (2.21)
Mean (SD)			

*MS*, multiple sclerosis; *RRMS*, relapsing–remitting MS; *PMS*, progressive MS

Checking the effect of different strategies of calculation of atrophy rates in SIENA (registration based detection of edge differences between timepoints) and SyMRI (two timepoint subtraction method), we calculated atrophy rates based on SIENAX results at the two timepoints, and calculated SIENAX PBVC using the subtraction method. The results are presented in the supplement. In brief, the between-subject variability of the two timepoint subtraction method (SIENAX PBVC) was considerably higher then in SIENA PBVC, and in tendency also higher than in PBVC calculated with SyMRI results (Table S3). The average change rates of Sienax PBVC were smaller and closer to zero atrophy than with Siena PBVC and SyMRI PBVC. Thus the subtraction method using SIENAX was less suited to estimate atrophy rates at this relatively short follow-up times, than SyMRI and SIENA.

## Discussion

In the present study, we validated the estimation of BPF from SyMRI in a large sample of patients with MS via comparison with the widely used SIENA(X) pipelines of FSL as the gold standard. In addition, we performed a longitudinal analysis of BPF measurement in patients with MS using SyMRI in a real-world clinical setting. High correlations between both methods suggested that they yield robust and comparable results. Furthermore good agreement highlighted the consistency of SyMRI compared with SIENAX segmentation. However, a systematic trend of higher BPF values estimated by SyMRI than SIENAX was seen, suggesting that SyMRI overestimated BPF, as well as GMV, relative to SIENAX. In contrast, SyMRI underestimated WMV and CSFV. Such overestimation of BPF and underestimation of CSFV was even more pronounced at higher BPF and lower CSFV values, reflecting excellent consistency (according to ICCs) in patients with brain atrophy and poor consistency in patients without atrophy. Therefore, it might be considered that the systematic bias of larger BPF estimation in SyMRI was mostly due to segmentation or partial volume effects of CSF, as this trend was less pronounced for WMV or GMV. As a secondary finding, we were able to show that larger GM volumes and smaller WM volumes calculated byn SyMRI compared to Sienax were not strongly dependent on the use of lesion-filled or non-lesion-filled T1w images, but must be due to other systematic differences between the methods.

Interestingly, in line with the present results, such overestimation of SyMRI for whole-brain and GM volumes was also obtained in a study comparing SyMRI with statistical parametric mapping (SPM) segmentation [35]. This study validated SyMRI volumetry in children by comparison with SPM and found consistent segmentation results, except for with CSFV, although a systematic influence of age and brain tissue volumes was observed.

Differences in brain volume measures between both methods could be due to the different segmentation algorithms. Because strong correlations and a good agreement between brain tissue volumes were observed between SyMRI and SIENAX, this systematic bias is likely the main source of the volume differences. The SyMRI segmentation approach is based on classification using relaxation times. The tissue types are related to the R1–R2–PD space using a predefined look-up grid, providing fractions of the tissue classes in each voxel [26]. In contrast, SIENAX uses a statistical segmentation approach in which the intensity and neighbouring sites influence spatial information [34]. Hence, differences in, for example, partial volume effects will possibly have an effect on brain volume estimates. Another reason for differences in brain volume is related to discrepancies in image resolution and geometry. Segmentation in SIENAX is based on an isotropic 3D T1w sequence with a slice thickness of 1 mm, whereas SyMRI segmentation uses a 2D image dataset with 4-mm slices. Thus, segmentation with SyMRI is more strongly affected by partial volume effects, which has been shown to be significant for volumetry in previous studies [26, 37, 38].

Because the main goal of developing automatic brain volume estimation approaches is to quantify disease-specific differences or therapeutic effects, it is of crucial importance to validate the ability to differentiate between disease types and to monitor longitudinal changes. Both aspects have been considered in this study. First, comparable reliability in differentiating between RRMS, PMS, and healthy controls was obtained for SyMRI and SIENAX. This confirmed the ability to quantify disease-specific brain volume loss using SyMRI segmentation, as it showed results consistent with the established SIENAX approach. For both methods, significantly lower tissue volumes were observed for PMS compared to RRMS and CS. Taking into account the duration of disease, which is significantly longer in PMS compared to RRMS, the volume differences were plausible from a pathophysiological point of view. The process of neurodegeneration and subsequent barin volume loss continues progressivly in the PMS group and intermittently in the RRMS group.

Second, in a smaller subset of patients with longitudinal data with relatively short follow-up times (median, 15 months), we compared brain atrophy rates assessed by SyMRI and SIENA. SyMRI showed higher atrophy rates than SIENA, approximately twice as high, both, in the whole sample and for each disease subtype. Considering the proposed cut-off value for pathological PBVC in MS of -0.4% [16], the present annualized PBVC value from SIENA of -0.47% for the PMS group reflected relevant brain atrophy within 15 months, slightly exceeding the cut-off, but this was not seen in the small RRMS sample (PBVC, 0.61%), which showed a very high variability of 1.5%. Comparable brain atrophy rates in PMS (about -0.5% / year) compared to RRMS have been reported previously in a study using SIENA for a similar follow-up period of 14 months [20]. Thus our SIENA results seem consistent with previous findings. Additionally, atrophy rates in the RRMS group might differ from PMS because our patients with RRMS had short to medium disease duration, while the disease duration in the PMS group was significantly longer. In contrast to SIENA, SyMRI yielded an averaged PBVC of -0.85% per year for PMS, corresponding to considerable brain atrophy rates compared to the -0.4% threshold, and 0.17% per year for patients with RRMS. These discrepancies between these methods complicate the interpretation and comparison of atrophy results and, thus provide evidence for the need to establish a method-dependent cut-off value for defining clinically significant brain atrophy rates. There are probably different effects that con contribute to higher brain atrophy estimations for SyMRI compared to SIENA especially in the PMS group. First it must be considered that there are methodological differences regarding the estimation of atrophy rates. Thus, SIENA uses a registration-based approach to estimate volume changes, whereas, for SyMRI, we calculated rates based on cross-sectionally obtained volumes from segmentation-based methods at each timepoint. Atrophy assessment using individually segmented MRI data is known to induce higher variabilities because, for example, differences in intensity scales are not taken into account and noise increases because of inconsistent segmentation of the same brain regions. Thus, segmentation-based methods are less reliable for longitudinal atrophy quantification in comparison with registration-based approaches [39]. Second, provided there is substantial brain atrophy during the follow-up period in the PMS group, the increase of the MS-lesion volume that is typically associated with atrophy development can lead to an additional loss of segmented brain volume in the follow-up MRI using SyMRI, as no lesion filling is used with this method and chronical MS lesions can partly be classified as CSF. Furthermore, more brain atrophy in the follow-up MRI and associated widening of sulci or ventricles might result in altered partial volume effects at the CSF-parenchymal boundaries, reflected by an additional loss of the segmented brain volume. Nonetheless, we observed in both methods, SIENA and SyMRI, consistent results regarding group-specific differences, similarly to the cross-sectional results. Thus, both approaches yielded higher atrophy rates for patients with PMS compared to patients with RRMS.

Due to the overestimation of brain volumes in patients with relatively high BPF, which applies particularly to the early phases of the disease, the results obtained with SyMRI have to be interpretated with caution in a clinical setting. Moreover, in future studies, more longitudinal data would be necessary to provide a method-dependent PBVC cut-off for SyMRI segmentation. An improved 3D SyMRI sequence that has recently been released for research purposes could be used to refine segmentation results at a higher resolution [40].

Overall, the automatic brain volume estimation based on SyMRI offers many advantages within a clinical setting. It requires less time than the FSL-SIENA(X) based approaches, not only for MRI acquisition but also for post-processing, as the SyMRI software can be used within the clinical PACS system without the need for image export and tissue volumes are calculated automatically. In contrast, SIENAX and SIENA require more post processing steps with user intervention, which have to be performed on external computers outside the clinical PACS.

### Limitations

Some limitations have to be considered when interpreting the present results. First, segmentations from SyMRI were not compared to manual segmentations but instead to the widely established reference methods from FSL. Although manual segmentation might be the true gold standard, it is elaborate and challenging and, therefore, causes high interrater variability. The aim of this study was to compare SyMRI segmentation with a proven and accepted method rather than determine its accuracy. Second, the present control group consisted healthy controls and patients who were examined to exclude any intracranial pathologies. Although these control patients did not show any radiological abnormalities in their brain MRI data, we cannot completely exclude differences in brain volumes in this control group compared with healthy controls. Therefore, the differences between patients with MS and control participants might have been underestimated or shown increased variability. In addition to that, the number of participants in the control group was relatively small compared to the patient groups. However, since our focus was a methodological comparison the group comparison to assess the performance of two different software methods rather than gain new insights in brain atrophy in MS, we think that the size of the control group was sufficient in this context. Third, the observation period of one year may be short, but we saw a benefit in observing whenerver or not an actual atrophy in a shorter period can be detected in a clinical setting, regardless of its scale, to provide potential measurements. Fourth, due to differences in slice thickness and segmentation (3D 1 × 1 × 1 mm resolution vs 2D 1 × 1 × 4 mm resolution), SyMRI is more strongly affected by partial volume effects.

## Conclusions

In summary, BPF estimations from SyMRI and FSL-based SIENAX or SIENA (for longitudinal analyses) are not interchangeable since an overestimation of volumes and brain atrophy rates, as well as higher variability of SyMRI results, were observed. However, the consistency and correlations between the two methods were satisfactory, and our results showed that SyMRI was similarly to FSL suited to quantifying disease specific atrophy in MS from both cross-sectional and longitudinal MRI. Therefore, our analyses provided evidence that SyMRI volume estimation delivered reliable results, and it can be considered a fast and easy-to-use alternative to tissue segmentation based on 3D T1w images.

### Supplementary Information

Below is the link to the electronic supplementary material.

Supplementary file1 (DOCX 539 KB)

## Data Availability

Patients’ raw MRI and clinical data cannot be made available due to data protection regulations. Other data can be shared on reasonable request to the corresponding author.
