# Supplementary material for: Automatic estimation of brain parenchymal fraction in patients with multple sclerosis: a comparison between synthetic MRI and an established automated brain segmentation software based on FSL
Source: Neuroradiology. 2023 Dec 18;66(2):193–205. doi: 10.1007/s00234-023-03264-0 (PMC10805841; doi:10.1007/s00234-023-03264-0)
Supplement: Supplementary file 1 — Supplementary file1 (DOCX 539 KB) [file 234_2023_3264_MOESM1_ESM.docx]

**Supplement to NRAD-D-23-00265:**

Yazici I et al. Automatic estimation of brain parenchymal fraction in patients with multple sclerosis: a comparison between Synthetic MRI and an established automated brain segmentation software based on FSL

**Comparison between SPMS and PPMS constituting the PMS group**

The PMS group consisted of N=23 patient with PPMS and N=84 patients with SPMS with similar BPF (table S1), age and EDSS. Mean (Standard deviation) of age and EDSS were for PPMS: 62 (9) years, 5.7 (2.2) and for SPMS: 56 (11) years, 6.1 (1.4). Thus, to achive similar group sizes in MS subgroups we pooled the PPMS and SPMS group to one PMS group for comparison with RRMS.

**Table S1** Comparison of BPF in RRMS, SPMS, PPMS and CS subgroups

|  | **CS** | **RRMS** | **SPMS** | **PPMS** | **p _ANOVA_** |
| --- | --- | --- | --- | --- | --- |
| BPF SyMRI | 0.86 (0.04) | 0.82 (0.05)^b^ | 0.75 (0.06)^a^ | 0.76 (0.05)^a^ | n.s. |
| BPF Sienax | 0.77 (0.02) | 0.75 (0.03)^b^ | 0.71 (0.03)^a^ | 0.72 (0.72)^a^ | n.s. |

ANOVA between MS subgroups and CS controlling for age, sex and disease duration. Pairwise post-hoc analyses using Bonferroni correction for multiple comparisons: a) Sinificant group difference between patient subgroup and CS, b) significant group differences between RRMS and SPMS

**Effect of lesion filling before running SIENAX on GM and WM volume results**

We assessed the effect of lesion filling, that is routinely applied before running SIENAX on 3D T1w images on the GM and WM volume results. In contrast to SIENAX, the SyMRI method does not include lesion filling and thus WM lesions are partly classified as GM.

To compare the effect of prior lesion filling on the SIENAX results, we ran the SIENAX analysis additionally on the original 3D T1w images without lesion filling. Omitment of lesion filling showed an overall small effect on GM and WM estimation by SIENAX (Table S2 and Fig. S1), without significant differences in GM or WM without lesion filling compared to the default analysis (paired t-tests: p>0.05 for WM and GM) .The systematic bias between SyMRI and SIENAX results that can be seen in the Bland-Altman plots (figure S2) remained mainly unaffected by the effect of lesion filling.

**Table S2.** Comparison of estimated grey matter volume (GMV), and white matter volume (WMV) using SIENAX (default lesion filling) and SIENAX without lesion filling

|  | GMV [ml] | WMV [ml] |
| --- | --- | --- |
| SIENAX | 576 (71) | 530 (58) |
| SIENAX without lesion filling | 574 (72)* | 531 (60)* |

* Comparison of means: p>0.05 in paired t-tests


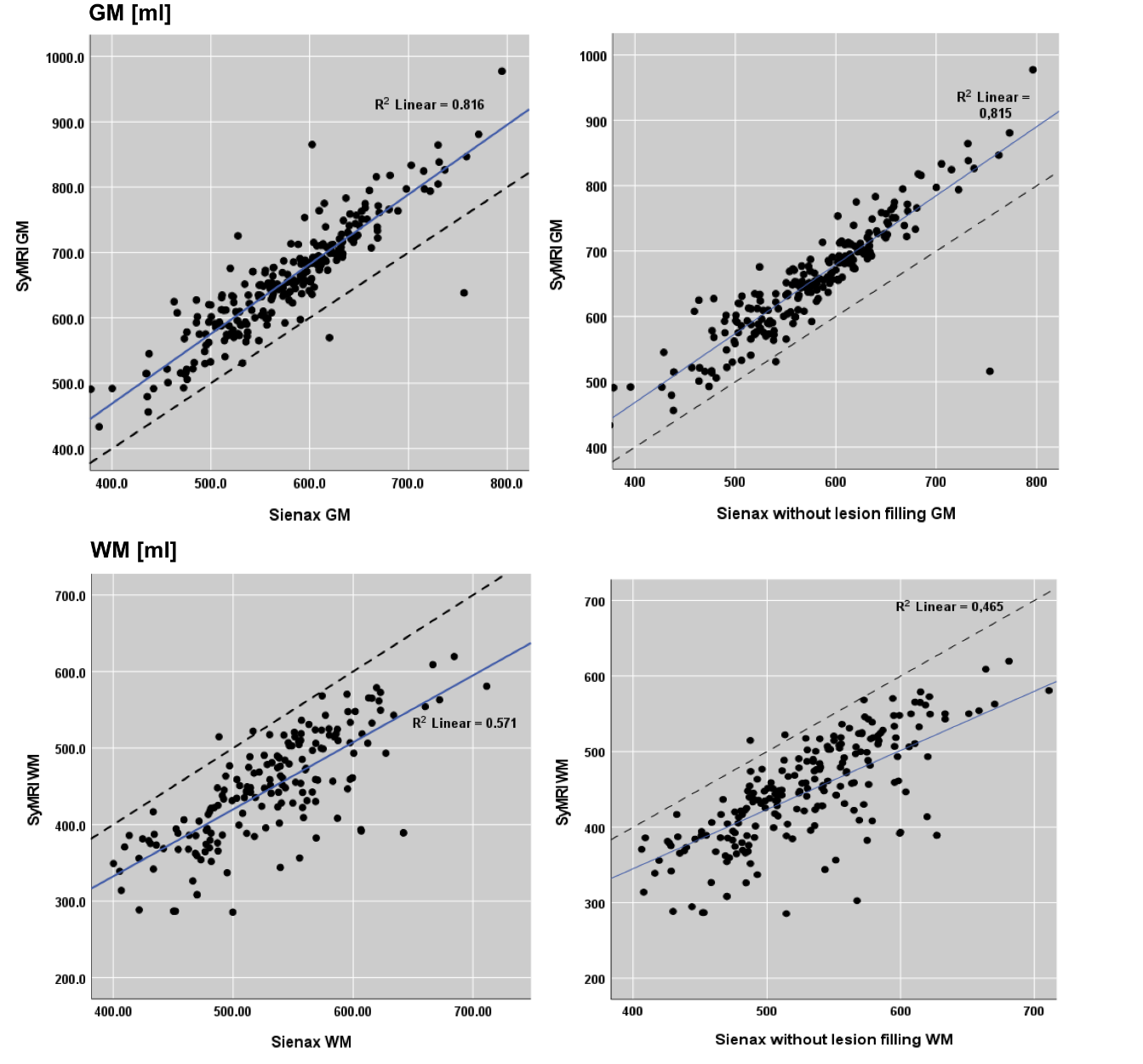
**Fig. S1** Comparison of the agreement between SyMRI and SIENAX outputs of GM and WM volumes in the entire participant groups with SIENAX being performed with or without lesion filling; upper row, left: scatterplot of GM by SIENAX (with lesion filling) over GM by SyMRI, right: scatterplot of GM by SIENAX (without lesion filling) over GM by SyMRI; lower row, left: scatterplot of WM by SIENAX (with lesion filling) over WM by SyMRI, right: scatterplot of WM by SIENAX (without lesion filling) over WM by SyMRI

**Comparison of WM and GM volume estimation by SyMRI and SIENAX in the control group**

The comparison of WM and GM volume estimation by SyMRI and SIENAX in the control group, where MS lesions are not present confirmed the systematic bias between the two methods independent of a potential lesion misclassification effect (Fig. S2).

**
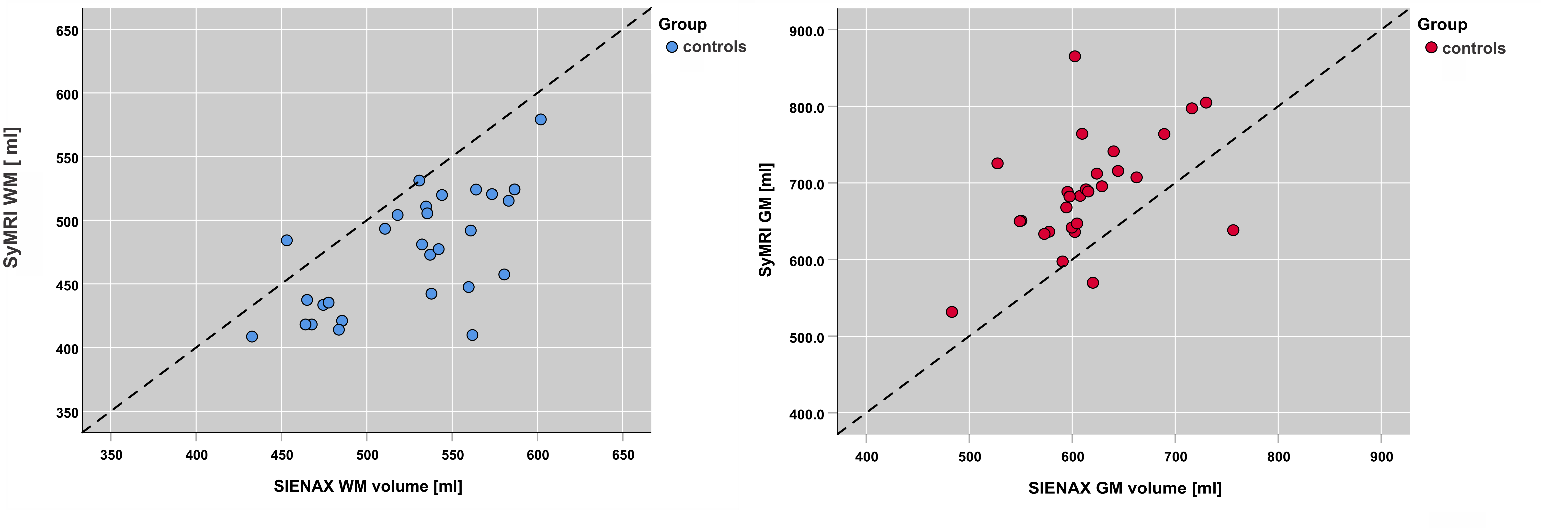
Fig. S2** Scatterplots of WM and GM SyMRI estimates plotted against SIENAX estimate in healthy controls; dashed line: identity line representing perfect agreement between the two methods.

**Longitudinal BPF change using Sienax at two timepoints.**

For a methodical comparison between two-point subtraction methods we calculated the percentual brain volume change (PBVC) for separate Sienax analyses at baseline (BL) and follow-up (FU) with the same formula as for the SyMRI data according to

$$\frac{BPF(FU)-BPF(BL)}{BPF(BL)}\times100$$

The between-subject variability of the two timepoint substraction method (SIENAX PBVC) was considerably higher then in SIENA PBVC, and in tendency also higher than in PBVC calculated with SyMRI results (table S2). The average change rates on Sienax PBVC were smaller and closer to zero atrophy than with Siena PBVC and SyMRI PBVC.

Thus calculating PBVC or aPBVC using the two-timepoint substration method with Sienax results was not suitable to detect brain atrophy in our data with a relatively short follow-up time.

**Table S3** Average percentage brain volume changes (PBVC) obtained by SIENA (registration based edge displacement method) and Sienax and SyMRI (two timepoint subtraction method). Annual atrophy rates were calculated using the time between two MRI examinations.

| Change rates [%] | Overall N=35 | PMS N=23 | RRMS N=12 |
| --- | --- | --- | --- |
| SIENA PBVC | -0.39 (0.84) | -0.65 (0.68) | 0.12 (0.89) |
| SIENAX PBVC | 0.052 (1.50) | 0.150 (1.43) | -0.137 (1.67) |
| SyMRI PBVC | -1.06 (1.39) | -1.44 (1.16) | -0.32 (1.55) |
|  |  |  |  |
| Annual |  |  |  |
| SIENA PBVC | -0.10 (1.09) | -0.47 (0.54) | 0.61 (1.50) |
| SIENAX PBVC | 0.147 ( 1.69) | 0.209 (1.51) | -0.027 (2.06) |
| SyMRI PBVC | -0.50 (1.52) | -0.85 (0.86) | 0.17 (2.21) |
| Mean (SD) |  |  |  |

MS, multiple sclerosis; RRMS, relapsing-remitting MS; PMS, progressive MS
